# A framework for characterizing heterogeneity in neurodevelopmental data using latent profile analysis in a sample of children with ADHD

**DOI:** 10.1186/s11689-022-09454-w

**Published:** 2022-08-03

**Authors:** Anne B. Arnett, Brian P. Flaherty

**Affiliations:** 1 Division of Developmental Medicine, Boston Children’s Hospital, Boston, MA 02115 USA; 2 Department of Pediatrics, Harvard Medical School, Boston, MA USA; 3 Department of Psychology, University of Washington, Seattle, WA USA

## Abstract

**Background:**

Heterogeneity in neurodevelopmental disorders, and attention deficit hyperactivity disorder (ADHD) in particular, is increasingly identified as a barrier to identifying biomarkers and developing standards for clinical care. Clustering analytic methods have previously been used across a variety of data types with the goal of identifying meaningful subgroups of individuals with ADHD. However, these analyses have often relied on algorithmic approaches which assume no error in group membership and have not made associations between patterns of behavioral, neurocognitive, and genetic indicators. More sophisticated latent classification models are often not utilized in neurodevelopmental research due to the difficulty of working with these models in small sample sizes.

**Methods:**

In the current study, we propose a framework for evaluating mixture models in sample sizes typical of neurodevelopmental research. We describe a combination of qualitative and quantitative model fit evaluation procedures. We test our framework using latent profile analysis (LPA) in a case study of 120 children with and without ADHD, starting with well-understood neuropsychological indicators, and building toward integration of electroencephalogram (EEG) measures.

**Results:**

We identified a stable five-class LPA model using seven neuropsychological indicators. Although we were not able to identify a stable multimethod indicator model, we did successfully extrapolate results of the neuropsychological model to identify distinct patterns of resting EEG power across five frequency bands.

**Conclusions:**

Our approach, which emphasizes theoretical as well as empirical evaluation of mixture models, could make these models more accessible to clinical researchers and may be a useful approach to parsing heterogeneity in neurodevelopmental disorders.

## Background

Heterogeneity in the genetic, neurocognitive, and phenotypic presentations of neurodevelopmental disorders (NDDs) is the focus of a growing body of literature in the fields of developmental pediatrics and psychology [1–5]. A potential advantage of parsing heterogeneity in NDDs is identification of homogenous subtypes that map onto precision medicine guidelines for clinical care. Attention deficit hyperactivity disorder (ADHD) is a strong candidate for this approach, due to the vast heterogeneity in biological and environmental mechanisms, high rates of co-occurring psychopathology, variable long-term outcomes, and multiple available pharmacological and behavioral treatment options [5–7]. Previous investigations have attempted to identify profiles that differentiate ADHD from control subjects utilizing neurobiological indicators and sophisticated analytic methods; however, classification accuracy is generally modest, and model adequacy may be biased due to sample size and the inherent heterogeneity within neurodevelopmental cohorts [8–10]. Accordingly, the current investigation aims to develop a framework for parsing heterogeneity *within* a neurodevelopmental or clinical cohort, using a case study of a small sample of children with ADHD and controls.

Symptom-based subtypes of NDDs, such as the predominantly inattentive versus predominantly hyperactive/impulsive clinical presentations of ADHD or Asperger’s syndrome versus autism or pervasive developmental disorder, have shown poor stability [11], interrater reliability [12], and inconsistent external validity [13]. Thus, subgroups defined by indicators that are distinct from the diagnostic symptoms themselves may present an opportunity to identify clinically relevant, cross-diagnostic features [7]. For example, a subgroup of individuals with a common neurobiological profile, despite variable NDD symptoms, may respond well to a specific treatment or be at increased risk for distinct adverse outcomes that could be addressed with preventative interventions. Consequently, there has been a shift toward identifying non-symptom-based subtypes of NDDs using exploratory clustering methods such as latent class analysis (LCA [14]), community detection [15], and regression trees [16], with neuropsychological [17], electroencephalography (EEG [14, 18]), event-related potential (ERP [19]), or psychiatric/behavioral [15, 20] indicators. However, the translational utility of these results is so far limited, due to a focus on single-method measurement (e.g., either neuropsychological or EEG variables, but not both) and the challenge of comprehensively characterizing clinical samples that are large enough to provide adequate statistical power for these models.

Common algorithmic clustering approaches, such hierarchical or k-means clustering, often lack a statistical model. These approaches ignore measurement error and “hard-classify” observations, which is to say these algorithms assign a case to a cluster or class with certainty. In contrast, statistical approaches to clustering, broadly referred to as mixture models [21], can model measurement error and classify probabilistically. Probabilistic classification accounts for the more realistic possibility that while some observations (i.e., individuals) may be likely to belong to a single class, others may be harder to classify. In mixture models, unless parameter restrictions are used or a solution includes boundary estimates, each observation will always have a nonzero probability of membership in each class, thus estimating classification uncertainty. Common approaches to mixture models, such as LCA or latent profile analysis (LPA), also present limitations in that they are considered large sample procedures. Particularly with small samples, it can be difficult to optimize the loglikelihood function and obtain proper maximum likelihood estimates because mixture models’ loglikelihood functions are frequently multimodal, meaning there are many local maxima. Practically, analysts run many sets of random start values to try to find a global maximum, as well as assess how well the parameters are estimated. Finally, LCA and LPA are frequently exploratory. Even when subgroups of observations are expected, the extant literature rarely provides sufficient information to fully characterize these classes ahead of time. Thus, confirmatory mixture models in clinical data are uncommon.

Fortunately, model fit and assessment of structural equation models (SEM) have been studied by applied statistics researchers for decades, and these procedures provide a road map for improved selection and evaluation of mixture models even in smaller samples [22]. Two general principles arise from the SEM literature. First, no single fit statistic or index is sufficient to evaluate a model; rather, multiple fit measures should be considered simultaneously. The second principle is that model evaluation and selection should first be guided by science and theory, not primarily by fit statistics [22, 23]. This emphasis on theory and science that we see in SEM has not been similarly adopted in applied mixture models, to date. Rather, the difficulty and subjectivity of interpreting and comparing plausible models are often side stepped with an appeal to parsimony, which translates to overreliance on the Bayesian information criterion (BIC [24]). While the BIC frequently performs well in mixture model simulation studies [25], it penalizes heavily for model complexity, compared to Akaike’s information criterion (AIC [26]), another common fit index used in mixture models. No statistical measure of fit, including AIC or BIC, has been found to be superior across research situations. Therefore, we propose that the principles identified in SEM (i.e., consider multiple criteria and heavily weight the science and interpretation) are the best current practice guidelines for evaluating mixture models in neurodevelopmental research.

In the current study, we aim to develop a statistical framework for parsing heterogeneity in neurodevelopmental disorders using LPA in sample sizes typical of neurodevelopmental research, i.e., < 150 subjects. As described above, one of the primary limitations of mixture models is the exploratory nature of the analyses, particularly with indicators for which there are no population normative values. Thus, we will begin by identifying a single-method model using indicators that have interpretable norms. Next, we will attempt to meaningfully interpret a second set of indicators (resting EEG data), by leveraging the structure identified in the single-method model. Consistent with procedures outlined in the SEM literature, we will examine all model aspects, rather than heavily weighting one or two indices of fit. Specifically, we will iteratively examine competing models by comparing their estimation quality, traditional fit indices, interpretation, measurement quality, and error variance. The obvious drawback to this holistic model selection approach is increased reliance on interpretation and subjectivity. However, as mixture models are typically utilized in the context of exploratory research to begin with, we argue that overreliance on fit indices risks rejection of potentially meaningful subgroups, which would be a disservice to clinical science in the long run.

## Methods

### Participants

One-hundred forty-one children ages 7–11 years were recruited from the greater Seattle, Washington area. Children were enrolled who had a clinical diagnosis of ADHD (*n* = 107) or did not have ADHD nor an immediate family history of ADHD (controls; *n* = 34). Exclusion criteria were diagnosis of intellectual disability, diagnosis of autism spectrum disorder, history of seizures, gestational age < 32 weeks, or prenatal exposure to drugs or alcohol. After enrollment, a subset of participants were excluded due to failure to meet diagnostic criteria for ADHD without autism (*n* = 15), elevated ADHD ratings in a control participant (*n* = 1), discovery of seizure activity during the EEG (*n* = 1), *IQ* < 80 (*n* = 2), or failure to abstain from psychotropic medications prior to the visit (*n* = 2).

The final sample included 30 controls and 90 ADHD participants, of whom 30% were assigned female at birth. The control group had higher full-scale IQ (range: 91–135, *M* = 118, *SD* = 10) than the ADHD group (range: 82–143, *M* = 107, *SD* = 12), *t* (56.33) = −4.42, *p* < .001. The groups did not differ on age (*t* [57.43] = 1.00, *p* = 0.319) or proportion of females (*χ*^2^ [1] = 0.053, *p* = 0.818). A significant minority of ADHD participants had elevated coexisting psychiatric symptoms according to caregiver ratings on the CBCL DSM-oriented scales (i.e., T score > 65): depressive problems (*n* = 32), anxiety problems (*n* = 14), somatic problems (*n* = 8), oppositional defiant problems (*n* = 35), and conduct problems (*n* = 27). Additionally, academic difficulties were indicated for a subset of ADHD participants, based on performance at least one standard deviation below average (i.e., standard score < 85) on the Wechsler Individual Achievement Test, 3rd Edition [27] single word reading (*n* = 17) or numerical operations (*n* = 10) subtests. One control participant had elevated oppositional symptoms, and one had below average performance on numerical operations.

### Procedures

Participants visited the University of Washington for a 3-h research visit that included parent-report questionnaires, 1 h of EEG, and 90 min of neuropsychological testing completed by a trained graduate student under supervision of a licensed clinical psychologist. Prior to the visit, children abstained from taking prescribed stimulant medications for at least 48 h, when applicable. Children taking other medications (e.g., non-stimulants) abstained for longer periods, according to guidance from their prescribing physician. ADHD diagnoses were confirmed by a licensed clinical psychologist specializing in pediatric ADHD using a combination of sources, including direct observation, caregiver report on the computerized version of the Kiddie Schedule for Affective Disorders and Schizophrenia [28], unstructured clinical interview, review of medical records, and/or caregiver report of at least six inattentive or hyperactive/impulsive symptoms on the strengths and weakness of ADHD and normative behavior DSM-5 ADHD checklist [29].

### Measures

#### Neuropsychological testing

Full-scale IQ (FSIQ) was derived from the two-subtest version of the Wechsler Abbreviated Scale of Intelligence, 2nd Edition (WASI-II [30]) and was thus separable from the remaining measures of executive functions and cognitive control. Measures of verbal memory, verbal and visual working memory, and orthographic processing speed were obtained from the digit-span forward, digit-span backward, picture span, and coding subtests of the Wechsler Intelligence Scale for Children, 5th Edition (WISC-V [31]), respectively. To measure variability of reaction time (VRT) and inhibitory control, participants completed a computerized stop signal task, administered on a laptop using E-Prime 2.0 [32]. The stop signal task consisted of 15 practice trials followed by 64 test trials, in which a continuous stream of visual stimuli (randomly ordered Xs and Os) was presented with a stimulus duration of 500 ms and interstimulus interval of 1030–1050 ms. Participants were instructed to press a corresponding key for each visual stimulus, except when presented with an auditory “stop signal.” The stop signal was a 1000 hz tone lasting 250 ms and was randomly presented prior to the onset of the visual stimulus on approximately 25% of trials. The duration of time between the stop signal and visual stimulus began at 250 ms and subsequently decreased or increased by 50 ms depending on whether the previous response was a correct inhibition or incorrect commission, respectively [33]. Stop signal reaction time (SSRT) was calculated as a measure of inhibitory control, using the subtraction method, which we determined was an unbiased approach given the low number of go-trial omission errors in our sample (median = 3.00; *IQR* = 1.00–7.00) [34]. VRT was calculated as the standard deviation of reaction time to go trials. Variables were normed and reverse coded as necessary such that higher scores indicated better performance.

#### Resting EEG

Participants were seated in a comfortable chair 70 cm from the presentation screen for the duration of the EEG. During the resting condition, children were instructed to sit quietly with their eyes open in a dark acquisition booth for four blocks of 30-s recordings, with brief (< 15 s) breaks between blocks, during which the lights remained off. After processing, the amount of data available for each participant ranged from 85 to 120 s for ADHD participants and 87–120 s for controls.

#### EEG recording

Continuous EEG was recorded with a high-density 128-channel Magstim-EGI Hydrocel geodesic sensor net and Net Station Acquisition software version 4.5.6 and 400-series high impedance amplifier (Magstim-EGI; Plymouth, MN). Signal-to-noise ratio was maximized by reducing electrode impedances to below 50 kOhms at the start of the session and by monitoring and rewetting electrodes throughout the EEG session. EEG signals were referenced to the vertex electrode, analog filtered (0.1 Hz high pass, 100 Hz elliptical low pass), amplified, and digitized with a sampling rate of 1000 Hz.

#### EEG processing

Continuous EEG data were processed offline using Matlab R2018b extensions and functions from EEGLAB 15 and ERPLab v8.0 packages. To aid with artifact detection and correction, initial processing of EEG data included an additional 3 min of baseline (lights on resting) EEG data and 30 min of ERP paradigms that were not analyzed as part of the current study. Data were downsampled to 250 hz and bandpass filtered at 0.3–80 hz. Electrical line noise from 55 to 65 hz was removed using the CleanLine plugin for EEGLAB. Bad channels were automatically detected and subsequently interpolated back into the dataset prior to average referencing, following methods outlined in [35]. Extended independent component analysis [36] with primary component analysis dimension reduction was used to identify and remove artifactual components (i.e., deriving from eye blinks, line noise, or cardiac signal) [37]. Continuous resting EEG data were fast Fourier transformed, and whole scalp absolute spectral power was extracted for delta (1–4 hz), theta (4–7 hz), alpha (8–12 hz), low beta (12–16 hz), and high beta (16–21 hz) frequency ranges. Values were then log transformed. Finally, power values within each frequency band were standardized within the sample prior to analyses to reduce computational load during the LPA and increase interpretability.

#### Analytic plan

**Fig. 1:**
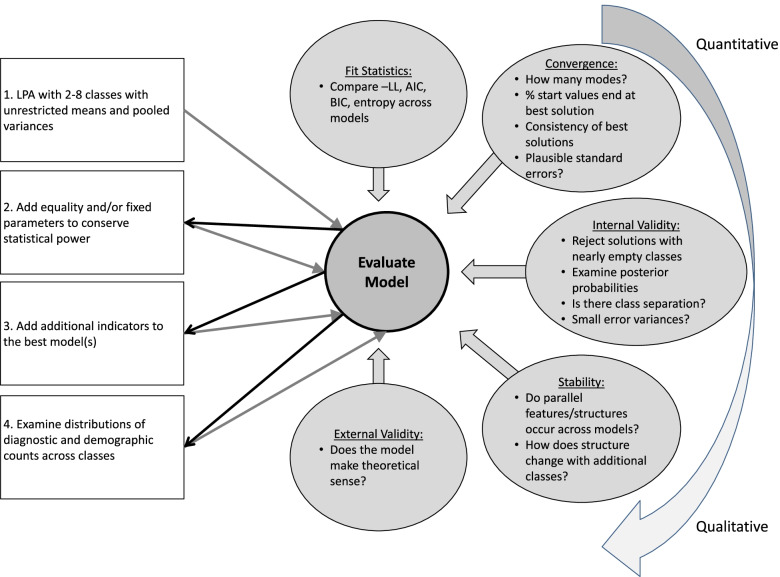
Flow chart depicting estimation steps for the single-method and multimethod latent models. Model evaluation metrics are described in the circles, ordered from the most quantitative (top) to the most qualitative (bottom) in nature

All raw item distributions were inspected, and one VRT outlier was winsorized from 446.88 to 346.88, which was near the next highest score. All data were standardized prior to analysis. LPA was conducted in Stata 17. Following the iterative framework illustrated in Fig. 1, initial analyses were done with neuropsychological variables. We chose to begin with neuropsychological indicators in this case study to demonstrate the utility of characterizing latent profiles that were separable from the diagnostic symptoms, unlikely to represent a continuum (i.e., low, medium, and high), and that were based on scores that were relatively understood with respect to population and ADHD norms. Moreover, neuropsychological evaluation is a frequent component of both research and clinical evaluations of NDDs, and while low performance on at least one test of cognition is common across NDDs, no single neuropsychological impairment fully explains variance in ADHD [38], nor do neuropsychological profiles reliably differentiate between ADHD and related NDDs [39, 40]. Thus, we expected to find heterogeneity in neuropsychological test performance even within our relatively homogenous ADHD sample.

At step 1 (Fig. 1, top left square), we began with unrestricted models specifying 1–8 latent classes, estimating each with 1000 sets of random start values. Next, at step 2, we considered (and ultimately rejected) the possibility of equating means across classes. Instead, we added parameter restrictions to specify two theoretically and data-driven classes and re-estimated models with 2–7 classes. That is, at this stage, the two-class model had two fully specified classes and only estimated one class proportion and seven pooled residual variances. The three- through seven-class models included the two fully specified classes, with the remaining classes having unrestricted means. Each of the parameter restricted models was compared with the previous unrestricted means models, as well as with one another. At step 3, we selected the best-performing model and tried adding EEG data, about which we had fewer hypotheses. We hoped to leverage the interpretability of the neuropsychological data to identify a stable model of EEG classes. Finally, we examined whether the classes differed on clinically relevant variables, such as demographics and ADHD diagnosis. At each stage, evaluation of model fit was comprehensive and included consideration of estimability, stability, interpretability, traditional model fit indices, and external and internal validity measurements (see Fig. 1, circles), which we describe in detail in the results.

## Results

### Step 1: single-method unrestricted means LPA

**Fig. 2:**
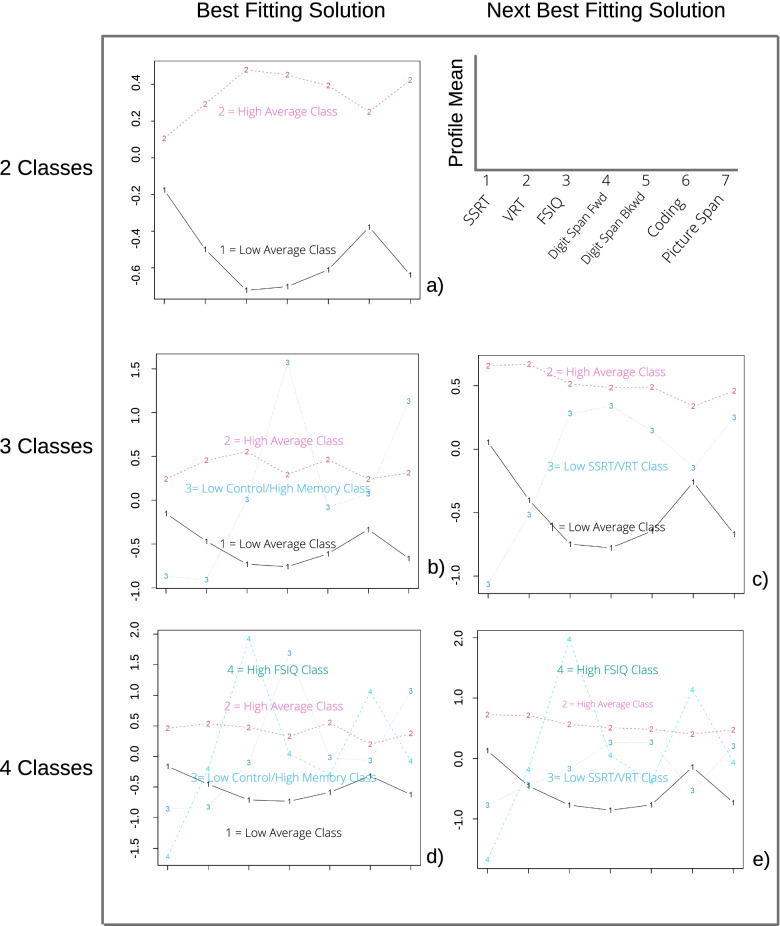
Plots of means for unrestricted models with 2–4 classes. The two-class solution converged on a single mode, while the three- and four-class models converged on multiple modes. The two best modes (i.e., with the highest loglikelihoods) are depicted. Profile means (y-axis) are standardized within the sample. SSRT stop-signal reaction time, VRT go-reaction time variability, FSIQ full-scale IQ, Digit Span Fwd digit span forward, Digit Span Bkwd digit span backward. Note recurring features in the data, such as the shape of class 1 (“low average”) across models, the shape of class 3 (“low control/high memory”) in the top three- and four-class models, and the shape of class 4 (“high FSIQ”) in the two best four-class models

We estimated unrestricted means LPA models with pooled variances, specifying 1–8 classes with all seven neuropsychological indicator variables. The one-class LPA had unacceptable fit statistics, as indicated by substantially higher AIC and BIC values, compared with larger models. This was an important result in our small sample size, because if the data were insufficiently powered to reject a model of independence, then we could not justify testing more complex models. The two-class LPA solution had a single mode (i.e., convergence on a single solution) that indicated low average (40%) and high average (60%) classes (Fig. 2a), the former of which we hypothesized was dominated by individuals with ADHD.

In contrast, the three-class loglikelihood function had four modes. All four of these three-class solutions included the low average and high average classes found in the two-class solution, indicating stability. However, the third class varied across modes. The solution corresponding to the best-fitting mode (i.e., with greatest −LL, Fig. 2b) included a third class with a relative weakness on measures of inhibitory and response control (SSRT and VRT) and a relative strength on measures of memory (digit span forward and picture span). This low control/high memory class comprised about 9% of the sample, an indication of internal validity. The second highest −LL three-class solution had a less extreme pattern but still captured individuals with relatively low SSRT and VRT (Fig. 2c). Next, we fit the four-class model, which had 11 modes, In the best-fitting four-class model, the low control/high memory class was retained, in addition to a fourth class with another interesting pattern that captured about 5% of the sample (Fig. 2d) and was characterized by the combination of above average FSIQ and coding, with low SSRT; we called this group “high FSIQ.” The next best four-class model included the three classes seen in the second-best three-class solution (low average, high average, and low SSRT/VRT) as well as the high FSIQ class from the best-fitting four-class model (Fig. 2e).

Compared to three- and four-class models, the five-class model had the most sets of random start values go to the best mode (Table 1); thus, it exhibited better convergence. The best-fitting five-class model included profile patterns corresponding to most of the classes discussed above in the best and second best three- and four-class models, indicating stability and lending credibility to the idea that these groups correspond to real subgroups in the data. Specifically, the five-class solution specified the low average, high average, low control/high memory, and high FSIQ classes. The new fifth class, which we called “below average,” was characterized by below average performance across all indicators except SSRT. The pooled residual variances for the indicators ranged from 44% for digit span forward to 86% for coding, indicating modest internal validity. The next best-fitting five-class model had substantially poorer fit (−LL = −1066.56) and was not considered.

**Table 1:** Fit statistics: unrestricted models with 1–8 classes

Number of classes	Parameters	Modes	Start values ending at top mode	LL	AIC	BIC	Entropy
1	14	N/A	N/A	−1144.41	2316.81	2355.84	1
2	22	1	100%	−1103.32	2250.63	2311.96	0.92
3	30	4	12%	−1090.29	2240.58	2324.21	0.91
4	38	11	10%	−1076.77	2229.53	2335.46	0.91
5	46	24	65%	−1063.67	2219.33	2347.56	0.90
6	54	50	9%	−1052.50	2212.99	2363.52	0.88
7	62	82	5%	−1040.98	2205.97	2378.79	0.89
8	70	115	0.1%	−1030.94	2201.87	2397.00	0.87

*LL* loglikelihood, *AIC* Akaike information criterion, *BIC* Bayesian information criterion, *N/A* not applicable

The six-, seven-, and eight-class models were not well estimated. Start values went to many more modes (≥ 50) with only minimally different loglikelihoods and fewer than 10% of the start values converging on the top mode. Investigation of the structures of the solutions revealed highly variable patterns of means. The six-class solutions included classes with variability in the low-performance end of the distribution, which is theoretically interesting; however, the exact patterns of means varied across the solutions, indicating poor estimation of these classes. Additionally, the seven- and eight-class models included classes comprised of a single individual in the sample (i.e., class prevalence ~1%), indicating poor external validity.

Altogether, we observed consistent features throughout the 2–5 class models. We closely examined shifts in class proportions as new classes were added and observed a high degree of stability. Specifically, as new classes were added, individuals tended to move out of the high average class into a new class with more variability across neuropsychological indicators (Table 2). As a result, the high average class developed a flatter profile as model complexity increased (i.e., as more classes were added). This consistency of class membership was another positive indicator of stability and possible validity of our models.

**Table 2:** Class proportions in recurrent classes across best-fitting 2–5 class models

	Low average	High average	Low control/high memory	High FSIQ	Below average
2 class	40%	60%	-	-	-
3 class	40%	52%	9%	-	-
4 class	42%	45%	8%	5%	-
5 class	46%	34%	9%	5%	6%

Column names correspond to classes observed across each of the 2–5 class models

### Step 2: Single-method LPA with restrictions

#### Equality restrictions

Having concluded that the three-, four-, and five-class models had the best model fit indices, we next imposed equality restrictions on indicator means across classes within each model. The goals of this step were to determine whether we could improve model fit by conserving statistical power, as well as to test stability of the classes. We began with the three-class model; based on estimates from the unrestricted three-class models, we restricted the means of VRT and coding to be equal across the low average and third classes (note, means of each indicator were equated across classes, not across all indicators). Likewise, we restricted the means of digit span forward, FSIQ, and picture span to be equal across the high average and third classes (again, means were equated only across classes, not indicators). This worked well in the three-class model, but the four- and five-class models showed poor estimation and stability. Thus, we decided to move forward without equality restrictions in our models.

#### Fixed parameters

An alternative to equality restrictions is to fix class item mean or variance parameters to a specific value. Based on results of the unrestricted models and our hypothesis that there would be a low-performing ADHD class and a higher performing control class, we added two sets of parameter restrictions. We specified two reference classes, with the first having means equal to −0.5 (i.e., one-half standard deviation below average) and the second having means equal to +0.5. By adding these restrictions, we reduced the number of parameters to be freely estimated by the models, thus increasing power to explain the remaining variance. At this stage, we examined models specifying 2–7 classes, based on favorable results returned for this range in the unrestricted models.

**Fig. 3:**
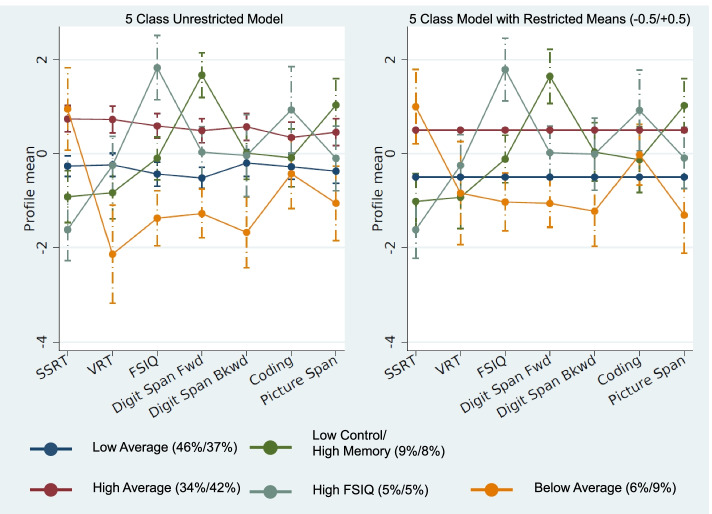
Plots of means and 95% confidence intervals for the unrestricted means (left) and fixed parameter (right) 5-class models. The fixed parameter model specifies a class with means of −0.5 (Low Average) and +0.5 (High Average). Numbers in parentheses are estimated proportions for each class (unrestricted/restricted). Note these values generally do not shift much, implying stability of the models. Profile means (y-axis) are standardized within the sample. SSRT stop-signal reaction time, VRT go-reaction time variability, FSIQ full-scale IQ, Digit Span Fwd digit span forward, Digit Span Bkwd digit span backward. Class proportions are depicted in parentheses (unrestricted model/restricted model)

The models with two fixed classes (i.e., one class with means fixed to +0.5 and one with means fixed to −0.5) showed improved estimability over the unrestricted models. The two- through four-class models had good convergence, with only one or two modes. Interestingly, both of the four-class modes were variations of means patterns that had emerged in the unrestricted models, increasing confidence that these were true features in the data and supporting use of a model that had greater than three classes. The five-class model had worse convergence, with six total modes, but a clear best solution (best solution, 567/1000 start values; substantially worse fitting solution, 418/1000 start values). In the best-fitting five-class model, the three freely estimated classes showed the same features seen in the unrestricted five class LPA (i.e., classes characterized by low control/high memory, high FSIQ, and below average; Fig. 3). Class distributions ranged from 5 to 42%, suggesting at least six individuals sharing a common item profile, even in the smallest class. Error variances were slightly worse in this model, ranging from 50% for digit span forward to 86% for coding. This was unsurprising, in that we were limiting the flexibility of the model to fit the data. Posterior membership probabilities were reasonably good in this model, with 74% of individuals with their highest class membership probability above 0.8.

The six-class LPA with two restricted classes was better estimated than the unrestricted six-class model, with 794/1000 start values going to the best solution. The best-fitting six-class model replicated results of the five-class model, with a sixth class characterized by indicator means falling approximately in the average range, between the two fixed classes, which was not theoretically interesting. In contrast, the next best six-class solution specified a sixth class that described approximately a single individual. Entropy for the best-fitting six-class model was poor, while AIC was lower and BIC higher than the five-class model. Finally, the seven-class model with two fixed classes was again poorly estimated and not considered.

Altogether, the five-class model with fixed parameters demonstrated a good balance across AIC, BIC, and entropy; the top mode fits considerably better than the second best mode, and the classes were both stable and theoretically interesting (Table 3). Thus, we opted to move forward with the fixed class five-class model in subsequent analyses.

**Table 3:** Fit statistics: 2–7 class models with parameter restrictions specifying means of 0.5 and −0.5

No. of classes	Parameters	Modes	Start values ending at top mode	LL	AIC	BIC	Entropy
2	8	1	100%	−1112.56	2241.125	2263.425	0.90207
3	16	1	100%	−1100.26	2232.511	2277.111	0.891765
4	24	2	77%	−1087.21	2222.418	2289.317	0.898637
5	32	6	57%	−1072.99	2209.973	2299.172	0.875514
6	40	17	80%	−1064.48	2208.956	2320.455	0.789589
7	48	33	0.8%	−1055.85	2207.702	2341.502	0.79027

*LL* loglikelihood, *AIC* Akaike information criterion, *BIC* Bayesian information criterion

#### Sensitivity analyses

As part of the second step in Fig. 1, we sought to further probe the stability of the results of the −0.5/+0.5 restricted five-class model by changing the means values for the reference classes. First, we tried shifting the reference means only slightly, to 0 and 1, respectively. The 0/1 restricted model had worse fit than the −0.5/+0.5 model; however, both models had structural features similar to those seen in the unrestricted five-class model, supporting stability of the −0.5/+0.5 solution. The only notable change was that the extremity of the difference in means between the low average and below average classes was attenuated relative to the unrestricted model.

A concern when using latent variable models with small samples is that there may be insufficient statistical power to reject a bad model [41]. Thus, we next examined the fits of two alternative restricted five-class models with extreme (and thus unlikely) reference classes: −1/+1, −2/+2, and −1/−2. In support of our ability to reject a bad model even with our relatively small sample size, estimability of these extreme fixed models was substantially worse. There were frequent estimation problems and failures to converge, which did not occur in the previous models. Additionally, fit statistics were far worse, and several classes had estimated proportions near zero. Thus, despite having a small sample, our ability to see differences in fit statistics across models suggested there was sufficient statistical power to capture reliable features of the data and unlikely to simply be modeling noise. As seen in Table 4, relying simply on AIC, BIC, or entropy is not sufficient. AIC and BIC values both favored the restricted −0.5/+0.5 model; however, we were not able to reject the extreme restricted models in favor of the unrestricted model using BIC alone. Likewise, entropy values were high for the extreme −2/+2 restricted model, because most individuals were captured in the freely estimated classes, with the reference class proportions nearing zero.

**Table 4:** Fit statistics: five-class model with fixed parameter restrictions

Model	Parameters	Modes	Start values ending in top mode	LL	AIC	BIC	Entropy
Unrestricted	46	24	65%	−1063.67	2219.33	2347.56	0.90
Restricted −0.5/+0.5	32	6	57%	−1072.99	2209.97	2299.17	0.88
Restricted 0/1	32	10	15%	−1079.39	2222.77	2311.97	0.86
Restricted −1/+1	32	9	2%	−1083.55	2231.71	2331.11	0.88
Restricted −2/−1	No convergence						
Restricted −2/+2	32	7	10%	−1090.29	2244.58	2333.78	0.90

*LL* loglikelihood, *AIC* Akaike information criterion, *BIC* Bayesian information criterion. Numbers in the restricted model names correspond to the values used to fix the means in two classes

#### Equality restrictions repeated

Having identified a relatively stable five-class model with two fixed classes (means = −0.5 and +0.5), we revisited the possibility of further conserving statistical power through equality restrictions. This time, we equated means of the VRT and coding indicators across the low control/high memory and high FSIQ classes. This model converged on two top modes (148/1000 and 143/1000 start values, respectively) which had comparable fit statistics. However, introduction of these equality restrictions resulted in significant shifts in means for these indicators, suggesting the models were contorting the solution to accommodate the equality restrictions. Thus, we did not include equality restrictions in future models.

#### Interpretation

To examine whether differences among the five latent classes were meaningful, we back-transformed the standardized indicator variables. Indeed, we found that differences among the classes were clinically interpretable. For example, the below average class scored at approximately the 9th–25th percentiles on tests of memory and processing speed. The high FSIQ class had a mean IQ at the 98th percentile but average performance (~50th percentile) on tests of memory and processing speed. The low average class had notably slower performance on the coding test of processing speed (16th percentile), with performance on other tests ranging from approximately the 25th to 50th percentiles. Finally, the low control/high memory class performed well above average on tests of memory (~91st percentile) but had relatively weaker performance on a test of processing speed (25th percentile). As depicted in Fig. 3, the 95% confidence intervals were largely nonoverlapping.

### Step 3: Add additional indicators to the model

Next, we aimed to go beyond the existing research by adding a second set of indicators to the model. In the current example, we added spectral power gathered during resting EEG, across five frequency ranges: delta, theta, alpha, low beta, and high beta. We hypothesized that by adding these lesser-understood indicators (i.e., brain data that do not have population normative values) to a model defined by indicators that were relatively well understood (i.e., neuropsychological test performance), we would increase the probability of identifying multi-method classes that were stable and theoretically meaningful.

#### Unrestricted multimethod model

First, we estimated a multimethod five-class model that included all seven neuropsychological and five EEG indicators. Parameter and equality restrictions were lifted, so that equal weight was allocated to all indicators. Results indicated that this unrestricted multimethod model was not well estimated; moreover, the EEG indicators overpowered the neuropsychological indicators, such that classes represented low to high EEG power and the structure of the five-class single-method model was entirely lost.

#### Restricted multimethod model

Next, we added restrictions so that we could estimate patterns of EEG indicator means corresponding to the five classes identified by the best-fitting neuropsychological model. We did not fix the means and variances of the neuropsychological indicators to match those identified in the final model because this would lead to improperly small standard errors and erroneous confidence in the model outcomes. Instead, we used multiple imputation with 20 imputed data sets. Individuals were randomly assigned to classes based on the posterior membership probabilities from the final neuropsychological model. For example, an individual with posterior probability of 0.90 for membership in the low average class would be included in that class during approximately 90% of the iterations; if this same individual had a posterior probability of 0.10 for the below average class, then their EEG values would be included in the below average class in about 10% of the iterations. Thus, the final estimate of means and variances for EEG powers within each latent class accounted for both class uncertainty and standard error of the sample means [42].

**Fig. 4:**
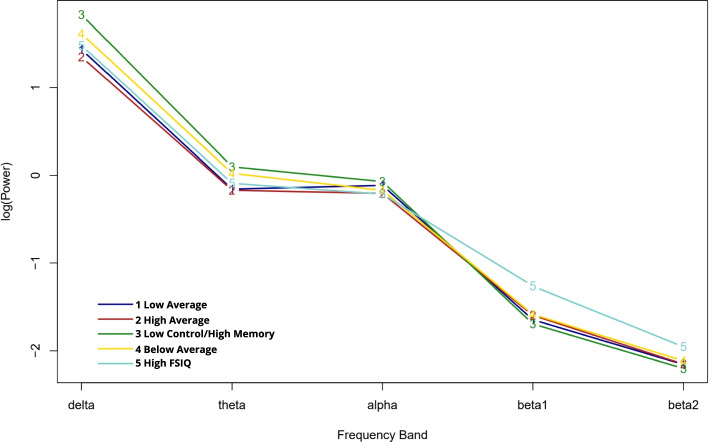
EEG spectral power profiles by neuropsychological latent class. Y-axis values are log-transformed absolute spectral power

The resulting EEG patterns are illustrated in Fig. 4. There was significant variability in EEG indicators for these classes. The low control/high memory and below average classes demonstrated elevated theta:beta ratio, which has previously been described as a potential neural signature of ADHD [43], while theta:beta ratio was low for the high FSIQ class (Table 5).

**Table 5:** Theta-beta ratio estimates by latent class

	Low average	High average	Low control/high memory	Below average	High FSIQ
Theta:beta 1	1.49 (1.43–1.55)	1.43 (1.37–1.48)	1.79 (1.57–2.01)	1.61 (1.37–1.84)	1.17 (1.01–1.33)
Theta:beta 2	2.00 (1.94–2.06)	1.98 (1.94–2.03)	2.30 (2.09–2.51)	2.14 (1.90–2.37)	1.86 (1.71–2.01)

Ratios were calculated for each individual using raw absolute power and then log transformed. Estimates for the classes were derived from multiple imputation with 20 data sets. 95% confidence intervals are reported in parentheses

### Step 4: Examine distributions of external variables across classes

To test the external validity and/or potential clinical utility of the multimethod model, the last step was to estimate counts, means, and variances of external variables of interest across classes, using latent class regression. First, we modeled sex and age as predictors simultaneously, because we did not have any a priori hypotheses about their association with the five latent classes. Sex was not significantly associated with any of the five classes (*p*s > 0.210). Older age was more strongly associated with the high average than the low average class (*B* = 0.48, *SE* = 0.24, *p* = .047, 95% *CI* = 0.01–0.95). No other effects of age were found (*p*s > 0.350).

We hypothesized that higher rates of ADHD participants in certain classes would add additional evidence of external validity for the model. Latent class regression failed because every member of the below average class was expected to be in the ADHD group. Thus, we again used multiple imputation with 20 iterations to estimate the proportion of ADHD versus control subjects in each class. ADHD diagnosis was highly probable in low average (88%), low control/high memory (70%), and below average (97%) classes and slightly less probable in the high FSIQ (34%) and high average (65%) classes.

## Discussion

### Overview

In the current study, we tested an iterative approach to estimating latent profile models using psychological data with a relatively small sample size. With high-quality, informative indicators (i.e., the neuropsychological data), our estimation process worked remarkably well. We were able to estimate, compare, and reject complex models using our small sample size largely due to estimation of the more complex models conditional on two reference classes (i.e., with means fixed at −0.5 and +0.5, respectively). By fixing parameter means in theoretically and data-driven classes, we conserved statistical power and leveraged remaining information in the data. As a result, we identified a five-class model that had strong convergence, high entropy, and moderate fit statistics; explained a high proportion of variance in the data; showed stability; and was theoretically interpretable. Critically, we may not have come to the same conclusion had we relied solely on BIC and AIC statistics, which are biased in small sample sizes.

Cluster modeling is often used in an exploratory context to better understand patterns of responses on a series of variables about which there are few a priori hypotheses. In the second part of our case study, we tested an approach in which we leveraged the stability of the five-class neuropsychological model (which had well understood indicators) to gain knowledge about less well-understood data (i.e., EEG spectral power). Not surprisingly, with twelve total indicators across the two domains, the five-class unrestricted neuropsychological + EEG LPA model was not stable, and did not retain the structure of the neuropsychological data. This is where we argue that the order of operations is particularly important, and that some subjective input may strengthen, rather than weaken, the outcomes of these models. Namely, we made a conscious decision to weight the structure of the five-class, neuropsychological indicator LPA with fixed classes more strongly than the unrestricted EEG data, by using multiple imputation to estimate means and variances of the EEG variables within each latent class. The result was that two classes showed interesting and theoretically plausible patterns of EEG spectral power; because these classes were small and the standard error of the EEG means was greater than those of the neuropsychological data, we would not have observed these patterns without first identifying the single-method LPA.

### Modeling guidelines

In this case study, we have provided an iterative flow chart to guide comprehensive evaluation of mixture models, similar to the recommendations made for SEM [22, 23, 41]. We propose that these guidelines will be particularly relevant to neurodevelopmental researchers who do not often have access to large sample sizes. Whenever possible, it may be useful to start with models that utilize indicators that are reliable and interpretable, to guide decision-making and provide a framework within which to gain knowledge about un-normed or exploratory data. The flow chart illustrates complementary qualitative and quantitative approaches to evaluating the estimability of the models at each stage. Although word limitations often impede documentation of model evaluation in the level of detail that we have provided here, we suggest that at the very least, choice of mixture models should be supported with qualitative as well as quantitative parameters. In the most qualitative recommendation, we advocate for using knowledge of the variables and extant literature to make a judgement about whether the model is theoretically sound. At the most quantitative end of the spectrum, we recommend comparing traditional fit statistics, like loglikelihood, AIC, BIC, and entropy. The suggestions in between (convergence, internal validity, and stability) are perhaps the most novel and yet provided some of the clearest guidance in the current case study.

It is a common practice for researchers to only examine the solution with the best loglikelihood and declare the solution reliable as long as it replicates an unspecified number of times. However, as we have demonstrated, examination of the number of modes and comparison of solutions across top modes (i.e., convergence) can be elucidating. Multiple modes with similar loglikelihoods indicate potential instability, while multiple nearby modes with differing solutions indicate a lack of convergence. Moreover, some investigation may reveal that the second or third best-fitting replicated mode may converge on a similar solution, suggesting greater stability than the top, unique mode. We noticed that in the three-class model, three modes replicated many times but revealed different solutions; yet, each of those solutions included features that showed up in subsequent models. This suggested that each mode was capturing aspects of a more complex underlying structure and gave us additional confidence in exploring a more complex model.

Rather than use an arbitrary class size cutoff for model selection, our internal validity check advocates examining models with small classes, within the bounds of reason for a particular dataset. For example, in our analyses, a class prevalence of 1% means about 1.2 people are predicted to be in this class. This seems like overextraction for this work. Yet, in a sample of 1000 people, a 1% class reflects about 10 people, which could be meaningful and consistent with the goal to fully explore patterns in the data. However, substantive interpretation must also be considered. A small class whose pattern of means differs only slightly from that of a larger class may not be necessary. On the other hand, a small class with a notably different and compelling profile of means may worth further exploration. In addition to theoretical interpretation, posterior membership probabilities for small classes should also be considered. Consistently, low posterior membership probabilities in a small class would suggest that this class does not capture any individuals’ response profiles well and is likely not worth retaining. On the other hand, even a small number of high posterior membership probabilities for a small class suggests that a small homogenous group has been identified. Of course, it is always possible that a small class is sample specific and will not replicate. However, we suggest that it is preferable to consider a small class and reject it later, rather than never seriously consider it. If one or more small classes do generalize and they are ignored, subsequent models are likely misspecified and will produce biased results. Furthermore, small classes may represent theoretically interesting behavioral patterns that could lend to highly tailored treatment plans.

### Model evaluation

Stability of the models can be examined in multiple ways. In the current study, we observed the same features in the data across models with different numbers of classes (e.g., the peak on FSIQ in the high FSIQ class), which suggested the models were capturing a subgroup of observations, rather than simply noise. Likewise, when we added restrictions to the data, the class structures did not shift much until the restrictions were highly incongruent with the data (e.g., fixed means at −2 and −1). Another way to evaluate stability is to add predictors to the model (in the current example, we used age and sex) and examine whether the class structures shift with a latent regression model.

### Case study results

With respect to interpretability of our final classes, we did observe features of the data that underscored our confidence in our approach. Specifically, the low average and below average classes are presented as consistent with a common clinical ADHD profile, with generally low or average performance across variables (with the surprising exception of SSRT in the below average class). In contrast, the high FSIQ class, which had a high probability of control participant membership, had above average IQ and average performance on most of the remaining variables. The high average class, which had means fixed to one-half standard deviation above average (i.e., +0.5), appeared to capture a proportion of controls as well as ADHD children who did not have a clear neuropsychological deficit. This phenomenon of children with behavioral, but not cognitive, symptoms of ADHD has previously been described [38]. Finally, the low control/high memory class emerged as a highly probable ADHD class characterized by variable reaction time and weaker inhibitory control (low scores on VRT and SSRT). Both of these measures have previously been identified as impaired in ADHD samples on average, as well as specifically within a subgroup of children with ADHD [17, 44]. Altogether, results of the single-method model demonstrated the cognitive heterogeneity often seen in neuropsychological profiles of ADHD [17, 45].

The patterns identified in the EEG data are likewise at least partially consistent with prior literature. Most notably, the low control/high memory and below average classes, which were high probability ADHD, were characterized by a high ratio of theta:beta band power [43, 46]. The fact that elevated theta:beta ratio was observed in two, but not all, of the high-probability ADHD classes is consistent with the extant literature, which finds inconsistent effect sizes for this neural signature [43]. In contrast, the high FSIQ class, of which 70% was estimated to be control participants, was uniquely characterized by high power in the beta frequency bands. The low average and high average classes had average EEG power across frequency bands, and their confidence intervals overlapped substantially with one another; thus, there is little to interpret in those groups. Importantly, with the exception of the delta and theta powers in the low control/high memory class and high beta power in the high FSIQ class, the standard errors of EEG power estimates overlapped across all five classes. This is not unexpected given the small sample sizes within each class and the greater degree of error in EEG as compared to neuropsychological data. An additional limitation is that power estimates were derived from the whole scalp, rather than specific regions, which likely increases standard error of these measurements [47]. Overall, there is additional probing to be done to better understand the association between the EEG outcomes and the neuropsychological profiles.

### Limitations

One of the most subjective steps in our estimation procedure, and that of any mixture model, was the selection of indicator variables. As already noted, we chose to start with neuropsychological variables because these measures are frequently used in clinical and research evaluations of children with NDDs and due to the availability of population norms to aid in interpretation of the scores. However, in ADHD and other NDDs, there are multiple sources of heterogeneity that could influence our outcomes and/or potentially be more reliable indicators, including co-occurring psychopathology (e.g., anxiety, depression), academic performance, and treatment response. In any case, an inherent limitation of our procedures is the reliance on known indicator variables and subjective interpretation of the results. The need for interpretation in our framework constitutes a barrier to discovering potentially meaningful subtypes derived from more exploratory measures, such as brain data.

Despite the promising theoretical outcomes and potential for further investigation, we must emphasize that these data were simply a case study used with the intent to test our hypothesis that with proper estimation techniques, it is possible to parse heterogeneity using small sample sizes and continuous data that are common in neurodevelopment. Our sample was small even for neurodevelopmental datasets, and thus, the results should be interpreted cautiously. We encourage researchers to attempt to validate their small study results in large, publicly available datasets, even if the indicator variables do not entirely overlap. Relatedly, it is important to recognize that the choice of indicator variables influences the models, and these measures will necessarily differ for neurodevelopmental samples that include children with lower cognitive, language, and/or adaptive functioning. In particular, several of the neuropsychological tests used in the current study rely heavily on the child’s receptive language abilities for valid administration, and thus do not adequately capture variance in the lower end of the cognitive distribution. Thus, these models require replication and further external validation before they can be interpreted from a broader neurodevelopmental perspective.

## Conclusions

We have demonstrated that a rigorous model evaluation process that simultaneously considers a range of quantitative and qualitative metrics can result in identification of a stable, complex mixture model in a small sample size. Novel and critical steps that maximized statistical power included specifying theoretically plausible parameter restrictions and starting with a set of indicators about which we had prior knowledge. We advocate for increased use of exploratory mixture models as a reasonable method for parsing heterogeneity in neurodevelopmental research and clinical samples more broadly.

## Data Availability

The datasets and analytic code used during the current study are available from the corresponding author on reasonable request.
